# Resolution in QCM Sensors for the Viscosity and Density of Liquids: Application to Lead Acid Batteries

**DOI:** 10.3390/s120810604

**Published:** 2012-08-03

**Authors:** Ana María Cao-Paz, Loreto Rodríguez-Pardo, José Fariña, Jorge Marcos-Acevedo

**Affiliations:** Department of Electronic Technology, University of Vigo, Campus Lagoas Marcosende, Vigo 36310, Spain; E-Mails: amcaopaz@uvigo.es (A.M.C.-P.); jfarina@uvigo.es (J.F.); acevedo@uvigo.es (J.M.-A.)

**Keywords:** quartz crystal microbalance, lead-acid batteries, state of charge, density-viscosity product measurement, sensitivity, frequency noise, resolution

## Abstract

In battery applications, particularly in automobiles, submarines and remote communications, the state of charge (SoC) is needed in order to manage batteries efficiently. The most widely used physical parameter for this is electrolyte density. However, there is greater dependency between electrolyte viscosity and SoC than that seen for density and SoC. This paper presents a Quartz Crystal Microbalance (QCM) sensor for electrolyte density-viscosity product measurements in lead acid batteries. The sensor is calibrated in H_2_SO_4_ solutions in the battery electrolyte range to obtain sensitivity, noise and resolution. Also, real-time tests of charge and discharge are conducted placing the quartz crystal inside the battery. At the same time, the present theoretical “resolution limit” to measure the square root of the density-viscosity product $\left(\sqrt{\rho \eta }\right)$ of a liquid medium or best resolution achievable with a QCM oscillator is determined. Findings show that the resolution limit only depends on the characteristics of the liquid to be studied and not on frequency. The QCM resolution limit for $\sqrt{\rho \eta }$ measurements worsens when the density-viscosity product of the liquid is increased, but it cannot be improved by elevating the work frequency.

## 1. Introduction

Lead acid batteries are among the most common types of batteries used in many applications such as electric vehicles, photovoltaic systems, remote stations, submarines, general support systems or telecommunications systems. With so many applications, it becomes essential at times to know the battery's real-time status in order to optimize equipment performance or to detect faults at an early stage. Ascertaining the state of the battery involves monitoring the current, power difference between the battery terminals, temperature, state of the charge (SoC) and state of health (SoH). SoC is one of the most important parameters to know because it enables improvements in the operation, performance, reliability and life span of the battery. Exceeding the manufacturer's charge limits may diminish the working life or even destroy the battery [1,2].

Quartz crystal resonators are piezoelectric devices which are used in electronic oscillators for a variety of purposes, because they can provide a highly accurate timing signal based upon the resonant frequency of the quartz [3]. If a mass is adsorbed or placed onto the quartz crystal surface, the resonant frequency and therefore the frequency of oscillation changes in proportion to the amount of mass. This property is widely used in research on mass adsorbed gasses, allowing quartz crystal oscillators to be used as high sensitivity microbalances intended to measure mass changes in the nanogram range by coating the crystal with a material which is selective towards the species of interest [4]. The name of these kinds of sensors is QCM or Quartz Crystal Microbalance. Also, from Kanazawa and Gordon [5] it is well-known that when a quartz resonator is in contact with a Newtonian liquid, there is a lineal relationship between the resonant frequency shift and the square root of the density-viscosity product of the liquid:

(1) $$\Delta f=k\cdot \Delta \sqrt{\rho \eta }$$

where Δf is the quartz crystal fundamental frequency shift due to the changes in the square of the density-viscosity product, $\Delta \sqrt{\rho \eta }$, and k is the sensitivity coefficient, given by (2):

(2) $$k=-\frac{2.26\cdot 10^{-6}f_{0}^{3/2}}{\sqrt{4\pi }}\frac{Hz}{\sqrt{\frac{g}{cm^{3}\text{poise}}}}$$

where f_0_ is nominal frequency of the sensor. In this way, a QCM sensor can be used to detect changes in the density-viscosity product of a liquid. Moreover, if the nominal frequency of the QCM sensor is increased, the sensitivity can be improved in order to measure the density-viscosity product changes in a tested liquid medium.

During the charge and discharge process in lead acid batteries, given that H_2_SO_4_ participates in the electrode reaction, the electrolyte becomes more diluted during discharge and is re-concentrated during charge [1]. Thus, parameters like density and viscosity vary according to the acid concentration present in the electrolyte. It is well-know that viscosity and therefore $\sqrt{\rho \eta }$ is more dependent on the sulfuric acid concentration than density [1]. Therefore, it is possible to use a QCM sensor to detect changes in the acid concentration by means of the frequency shift due to the changes of the square of the density-viscosity product.

This paper describes the characterization and application of a 9 MHz Miller QCM oscillator sensor for real-time monitoring of the SoC during charge and discharge processes of a lead acid battery [6,7]. Also, a new expression for the resolution limit that can be achieved with QCM sensors to measure changes in the density-viscosity product of a liquid load is obtained in the theory section. This expression completes the resolution limit theory previously published by Rodriguez-Pardo *et al.*, in [8] with the case of QCM sensors for physical properties of fluids.

The main text is divided into four sections. In the first section, resolution theory is developed and temperature considerations are discussed. In the second section, the experimental set-up is described: calibration and characterization of the sensor with sulphuric acid solutions in the battery range, thermal characterization, home-made quartz holder for placing the resonator inside a battery cell, and real-time tests of the sensor in charge and discharge process. The third section describes results and discussions, and the fourth and final section summarizes the conclusions. It will be shown that the sensor is able to detect changes in the battery's SoC of about 0.2% with a time interval of 2 s.

## 2. Theory

In order to characterize a QCM sensor, it is necessary to determine not only sensitivity but also the oscillator's frequency stability and its resolution. The frequency stability of an oscillator is its capacity to maintain the frequency of the output signal constant with time. The study of the frequency noise in QCM oscillators is very important: it allows the determination of the lowest possible frequency shift of the electric signal that is can be detected due to the mass or liquid variation to be established [8].

To estimate frequency stability in the time domain, it is necessary to determine the dispersion of a set of frequency measurements. Normally, classical statistics such as standard deviation or variance are used to measure dispersion. However, variance works only with stationary data, where the results must be time independent and the oscillator frequency is usually nonstationary. For this reason, nonclassical statistics are used to estimate frequency stability in the time domain. For a normalization of the frequency stability measurements in the time domain, the IEEE has proposed the Allan deviation of Equation (3) [9]. The oscillator detection limit can be estimated using this deviation Equation (4), *i.e.*, the smallest frequency deviation that can be detected in presence of noise. The resolution of the sensor can then be obtained using the relationship between the detection limit and the sensitivity coefficient Equation (5) [8]:

(3) $$\sigma_{y}^{2}(m,\tau )=\frac{1}{2f_{o}(m-1)}\sum_{n=1}^{m-1}{\left({\bar{f}}_{n+1}(\tau \right)-{\bar{f}}_{n}(\tau ))}^{2}$$

(4) $$\Delta f(\tau )=\sigma_{y}(\tau )\cdot f_{o}$$

(5) $$\text{Resolution}=\frac{\Delta f(\tau )}{k}$$

(6) $${\bar{f}}_{n}(\tau )=\frac{1}{\tau }\int_{t_{n}}^{t_{n}+\tau }f(t)dt$$

where *f̄_n_*(τ) (from now on *f̄_n_*) is the nth sample of the average frequency calculated over a time interval *τ* starting from an instant *t_n_* Equation (6), *f_o_* is the nominal frequency of the sensor, *m* is the number of samples and *k* is the sensitivity coefficient.

### 2.1. QCM Resolution Limit to the Measurement of $\sqrt{\rho \eta }$

Vig and Walls proposed an empirical relationship between the smallest level of noise generated by an oscillator in vacuum and the quality factor of the resonator, Q, for sampling times, t, between 0.1 and 10 s [10]:

(7) $$\sigma_{y}{\left(\tau \right)}_{\min}=\frac{1.0x10^{-7}}{Q}$$

**Figure 1.**
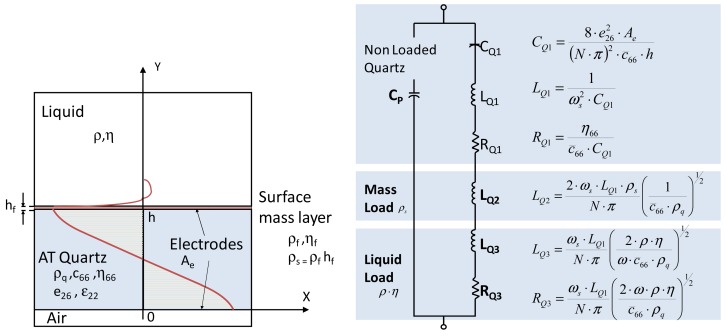
Electromechanical model of a piezoelectric resonator for microgravimetrical applications in liquid; Cross-section of a loaded resonator and BVD equivalent circuit modified by Martin and Granstaff.

Equation (7) indicates that frequency noise decreases when quality factor increases. For QCM sensors working in liquids, the resonator damping degrades the maximum quality factor achievable. Figure 1 shows the resonator equivalent circuit proposed by Van Dyke and Butterworth [11] and modified by Martin & Granstaff [12] taking into account mass and liquid load.

When there is no mass loading, L_Q2_ = 0. Usually L_Q3_ ≪ L_Q1_, and assuming ideal quartz (R_Q1_ ≈ 0), then the maximum Q for the resonator in liquid is given by Equation (8) [8], where $\bar{c_{66}}$ is the shear stiffness constant of the quartz and ρ_q_ is the quartz density:

(8) $$\begin{array}{c} Q_{\max}\approx \frac{1}{\sqrt{f_{0}}}\sqrt{\frac{\bar{c_{66}}\rho_{q}\pi }{4}}\frac{1}{\sqrt{\rho \eta }}\\ Q_{\max}\cdot \sqrt{f_{o}}\cdot \sqrt{\rho \eta }\approx (7.8x10^{5})\frac{g}{cm^{2}\cdot s} \end{array}$$

Then, the minimum attainable frequency noise, supposing Q_max_, increases with the frequency and with the product ρη:

(9) $$\sigma_{y}{\left(\tau \right)}_{\min}=1.28\cdot 10^{-13}\sqrt{f_{0}}\sqrt{\rho \eta }$$

In this case, the best oscillator detection limit to density-viscosity changes is given by:

(10) $$\Delta f_{\min}=1.28\cdot 10^{-13}f_{0}^{3/2}\sqrt{\rho \eta }HZ$$

According to Equation (5), the best resolution is calculated by dividing the minimum detection limit by the sensitivity coefficient, k, given in Equation (2). It results in the expression:

(11) $$\text{Resolution}=2x10^{-7}\sqrt{\rho \eta }\sqrt{\frac{g}{cm^{3}}}cp$$

The equation obtained for the resolution indicates that the best resolution to the liquid parameters (ρη) that can be obtained with a QCM sensor does not depend on the oscillator frequency but does depend on the liquid characteristics.

### 2.2. Temperature Considerations

Real-time battery applications produce changes in the temperature of the electrolyte during charge and discharge processes. These changes will cause variations in the density-viscosity product of the liquid, resulting in a resonant frequency shift of the QCM sensor. Likewise, quartz experiences frequency resonance thermal drifts inherent to crystal behavior [10,13]. In order to compensate these two effects, the temperature coefficients of the QCM sensor have to be obtained [14].

Changes with temperature in the density of the electrolyte in lead acid batteries are well-known values. Also, the Arrhenius and Poiseuille equations predict the changes in the viscosity of a solution with temperature. A solution of 40% sulfuric acid corresponding to a fully charged battery has the following expressions for the density and viscosity drifts with temperature [15,16]:

(12) $$\Delta \rho (T)=-7.45\cdot 10^{-4}(T-T_{0})$$

(13) $$\Delta \eta (T)=-1.53\cdot 10^{-5}{\left(T-T_{0}\right)}^{3}+1.02\cdot 10^{-3}{\left(T-T_{0}\right)}^{2}-4.98\cdot 10^{-2}(T-T_{0})$$

where T_0_ = 25 °C is the reference temperature. On the other hand, the thermal drift of an AT-cut quartz resonator can be expressed as in (14):

(14) $$\frac{\Delta f(T)}{f_{0}}=A_{1}[T-T_{0}]+A_{2}{\left[T-T_{0}\right]}^{2}+A_{3}{\left[T-T_{0}\right]}^{3}$$

where A_1_, A_2_ and A_3_ are the static temperature coefficients and T_0_ is the temperature of reference (typically T_0_ = 25 degrees Celsius). Some authors give values for the AT-cut temperature coefficients of the quartz [17–20]. However, it is preferable to estimate individual coefficients for each application [21]. In next section thermal coefficients are obtained for a fully charged battery.

## 3. Experimental Section

**Figure 2.**
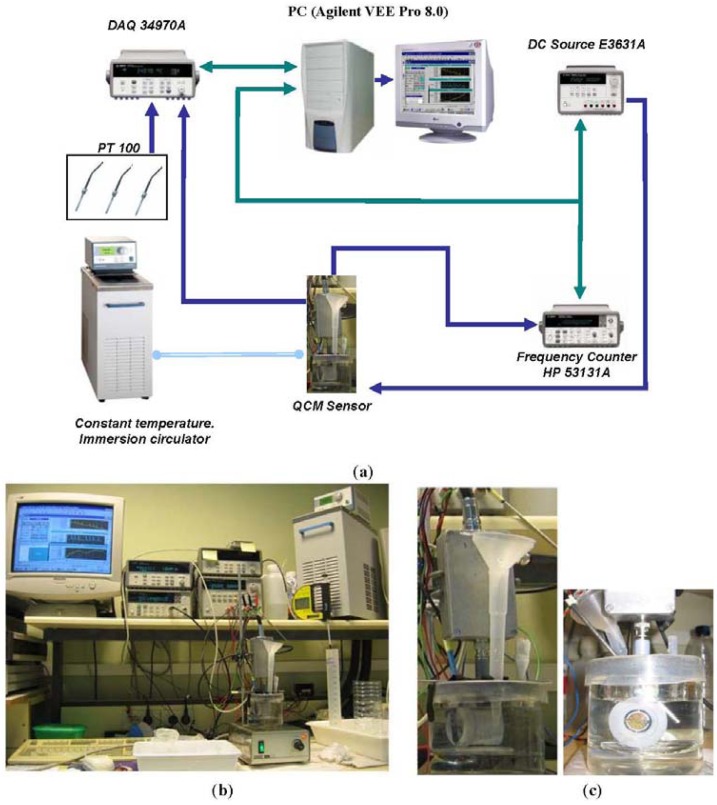
Experimental setup for the calibration of the sensor (**a**) Connection diagram, (**b**) Experimental test bench, (**c**) Quartz Crystal during the calibration with solutions of sulphuric acid.

A home-made 9 MHz Miller oscillator was used as SoC QCM sensor for lead acid batteries [6,7]. In order to estimate its experimental sensitivity coefficient to changes in the density-viscosity product of the electrolyte, calibration was performed with the quartz resonator immersed in sulfuric acid solutions thermostated at 30.8 °C using a PolyScience Heating Circulator. The temperature of the electronic oscillator was maintained stable at 42 °C during the calibration using a Watlow heater monitor. The sulfuric acid concentration in the solution was changed in steps from 13% to 40%, which corresponds to a change of 13% to 99% in the state of charge of a battery. To do this, small amounts of concentrated sulfuric acid (95%) were added periodically to the solution in order tosimulate the battery charge process. Figure 2(a,b) shows the equipment used and a connection diagram of the test bench, respectively. A detail of the QCM sensor placement is shown in Figure 2(c). The oscillator frequency and the solution temperature were monitored throughout the whole process with a sampling time of 2 s. Density measurements of the solution were also made for each sulfuric acid concentration step with an Anton Paar DMA-35 precision density meter. Parameters of the equivalent circuit of the resonator were also measured for each concentration with an HP-4194A impedance analyzer.

With the aim of knowing the detection limit (*i.e.*, the minimal frequency variation (Δf) that it is possible to measure due to changes in $\sqrt{\rho \eta }$), a study of the short term stability was carried out. Allan deviations for each concentration of sulfuric acid in the solution were calculated using 150 samples with a sampling time of 2 s.

In order to obtain the thermal compensation coefficients for the SoC QCM sensor, a test with the resonator immersed in a 40% of sulfuric acid solution was carried out. The temperature of the solution was changed between 17 °C and 25 °C, and the temperature of the electronic oscillator was maintained stable at 42 °C. The oscillation frequency, the temperature of the solution and the temperature of the oscillator were monitored throughout the process with a sampling time of 2 s. Using the equations for variation of density and viscosity of the solution with temperature (Equations (12) and (13)) and the Kanazawa and Gordon equation of frequency variation with the square root of the density-viscosity product (Equations (1) and (2)) for a nominal frequency of 9 MHz, that part of the frequency shift due to the contribution of the temperature in the density-viscosity product of the solution was determined:

(15) $$\Delta f_{\rho \eta }(T)=4.92\times 10^{-6}{\left(T-T_{0}\right)}^{3}-4.33\times 10^{-4}{\left(T-T_{0}\right)}^{2}+3.19\times 10^{-2}(T-T_{0})$$

**Figure 3.**
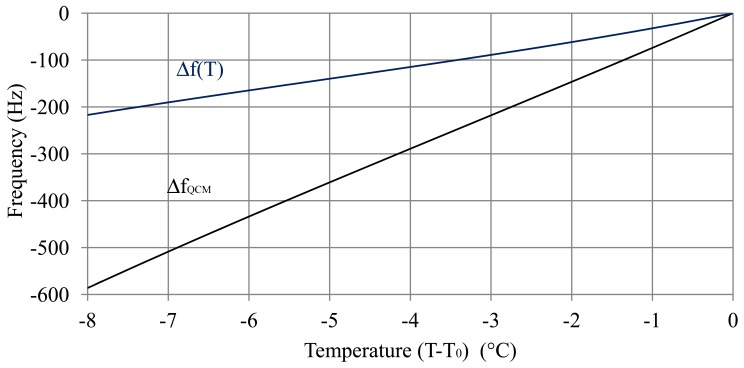
Variation of the oscillation frequency with temperature in a fully charged battery (40% sulphuric acid), Δ*f_QCM_*, and compensation of the variation of the square root of the viscosity-density product with temperature, Δf(T) = Δ*f_QCM_*−Δ*f_ρη_*(T).

Figure 3 shows the frequency variation obtained during the thermal test (Δ*f_QCM_*) and the frequency shift after compensation of the variation of the square root of the density-viscosity product with temperature by using Equation (15):

(16) $$\Delta f(T)=\Delta f_{\text{QCM}}-\Delta f\rho \eta (T)$$

Using the curve Δ*f*(*T*) of Figure 3, the thermal coefficients were obtained for the quartz in the electrolyte (Equation (14)):

(17) $$\begin{array}{c} A_{1}=3.7\times 10^{-6}\frac{1}{{}^{\circ}\text{C}}\\ A_{2}=198\times 10^{-9}\frac{1}{{\left({}^{\circ}\text{C}\right)}^{2}}\\ A_{2}=12900\times 10^{-12}\frac{1}{{\left({}^{\circ}\text{C}\right)}^{3}} \end{array}$$

**Figure 4.**
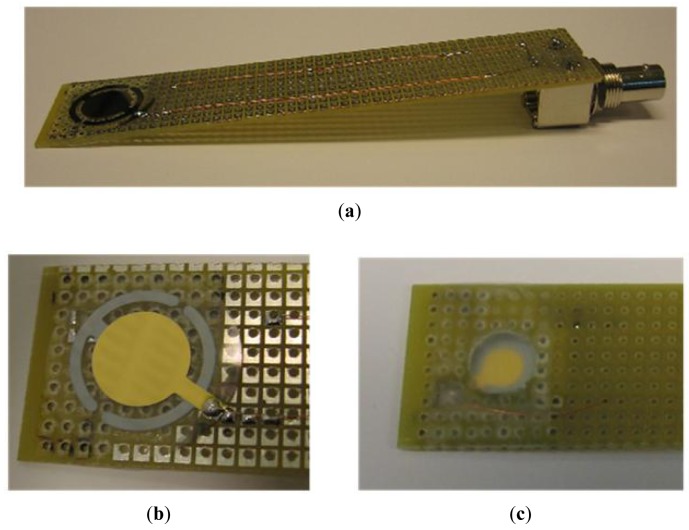
Quartz crystal holder designed for real-time battery measurements. (**a**) Appearance of the crystal assembled on the holder; (**b**) Detail of crystal area; (**c**) Air chamber for crystal oscillation.

Later, the sensor was tested inside a lead acid battery. A suitably shaped holder had to be designed to place the quartz crystal inside the cell through the ventilation holes in the top of the battery (Figure 4(a,b)). This holder includes an air chamber to allow for the oscillation of the crystal (Figure 4(c)). In addition, an insulation coating was used to protect the holder and the quartz crystal, except for the face in contact with the battery electrolyte. The insulation coating's purpose is to provide electrical insulation and waterproofing.

**Figure 5.**
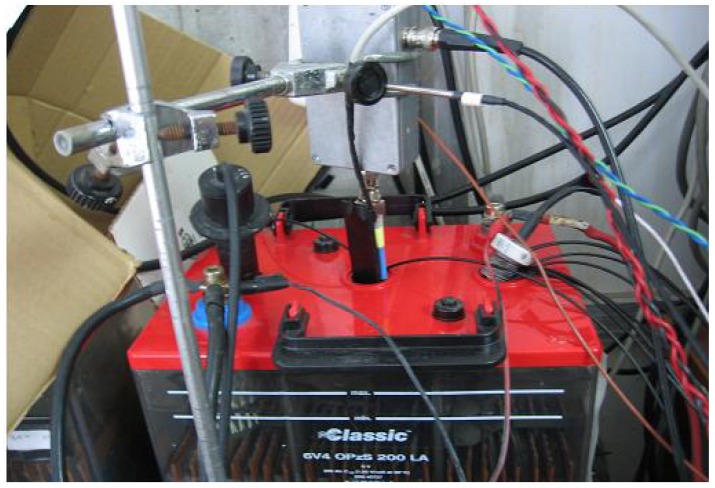
QCM sensor placed inside the battery during real time measurements.

A study of short term stability was carried out with the sensor inside the battery, given that the operating conditions of the quartz are different from those in the controlled trial with sulfuric acid solutions. Inside the battery, the environment can be turbid due to suspended particles. This can produce instabilities in the sensor. In order to estimate this effect and any other that could produce frequency noise, it is desirable to know the resolution of the sensor inside the battery. Moreover, after thermal compensation, it is interesting to know the frequency noise. The resonator was placed inside a battery cell in the top area of a battery with a SoC of 99% (Figure 5). The temperature of the electronic oscillator was maintained constant at 42 °C, but the electrolyte was at room temperature (temperature of the battery). Oscillation frequency, temperature of the electrolyte and temperature of the electronic oscillator were sampled every 2 s over four days. Temperature compensation was carried out and Allan deviation and resolution were determined in real conditions for application of the SoC QCM sensor.

Finally, the sensor was tested in real-time charge and discharge processes. Charge and discharge trials were carried out with the quartz inside the battery. Once again, oscillation frequency, oscillator temperature and temperature of the battery were monitored every 2 s, and a temperature compensation of the measured frequency was carried out in order to obtain the variation of the density-viscosity product in the battery and therefore the variation of the SoC with time in both charge and discharge processes. Also, the density of the electrolyte was periodically measured with an Anton Paar DMA-35 precision densimeter in order to check the good correlation between SoC and QCM measurement.

## 4. Results and Discussion

### 4.1. Calibration in Sulfuric Solutions. Sensitivity, Frequency Noise and Resolution

**Figure 6.**
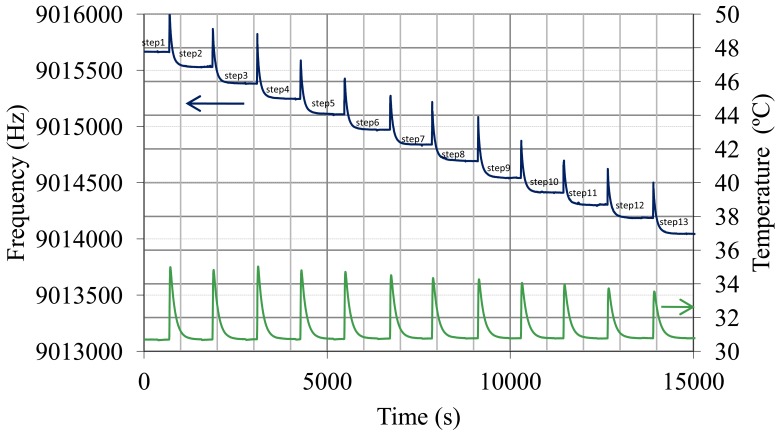
QCM sensor frequency and liquid temperature during solutions test.

**Figure 7.**
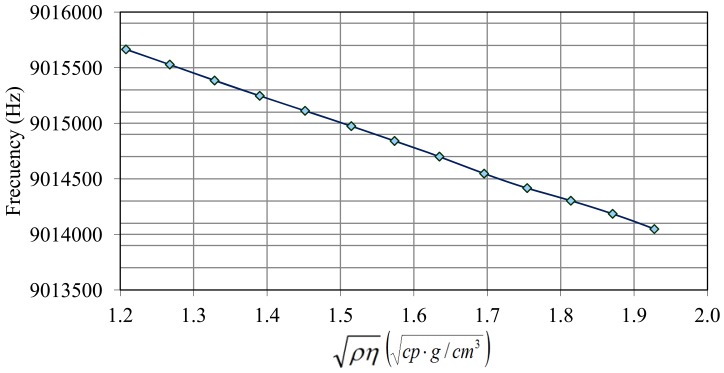
Calibration plot of the QCM sensor.

With respect to the calibration with sulfuric solutions, Figure 6 shows both the frequency changes when concentrated acid is added and the liquid temperature. Frequency has some ascending peaks which correspond to pure acid addition. This action produces an exothermic reaction and frequency is affected by temperature increment. A few minutes later liquid temperature returns to its stable value (30.8 °C) and the frequency shift responds only to the square root of the density-viscosity product. Taking the values of frequency and density measurements in areas where the temperature becomes constant again (linear zones of frequency steps in Figure 6), the calibration plot in Figure 7 was obtained, and an experimental sensitivity $K_{\text{experimental}}=\Delta f/\Delta \sqrt{\rho \eta }$ of $-2.2\text{kHz}/\sqrt{\frac{g}{cm^{3}}}cp$ was determined for this sensor. The theoretical sensitivity coefficient calculated by Equation (2) is $K_{\text{theoretical}}=-1.7\text{kHz}/\sqrt{\frac{g}{cm^{3}}cp}$.

**Figure 8.**
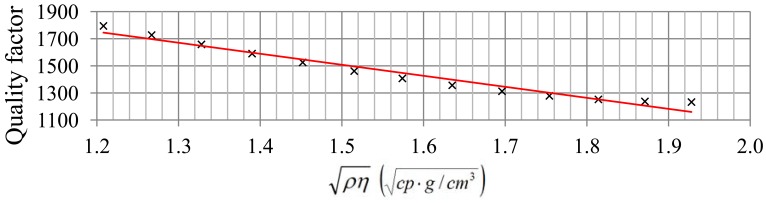
Relation between quality factor and $\sqrt{\rho \eta }$.

**Figure 9.**
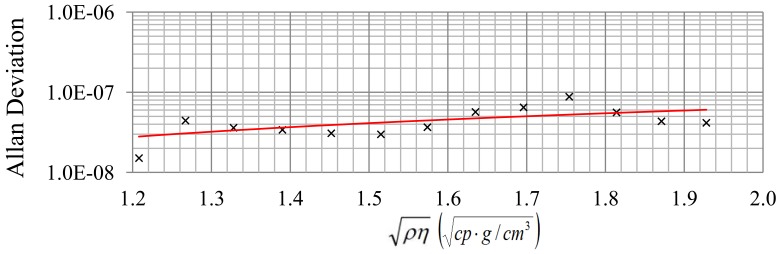
Relation between Allan deviation and $\sqrt{\rho \eta }$.

In Table 1, the experimental quality factor, Allan deviation, detection limit and resolution obtained for each pair $\sqrt{\rho \eta }$ (each frequency step in Figure 6) are indicated. These results show that the noise is higher than the minimum attainable using Equation (9) perhaps because of problems of circuitry. The resolution decreases slightly when $\sqrt{\rho \eta }$ increases, because quality factor and therefore noise tendency becomes greater with $\sqrt{\rho \eta }$ as expected for a given nominal frequency. Figures 8 and 9 show this tendency. The greatest value of the Allan deviation (8.8 × 10^−8^) represents the worst case. In this situation, the experimental quality factor, Q_exp_, is 1,279 and a resolution of $4.6\cdot 10^{-4}\sqrt{\frac{g}{cm^{3}}cp}$ can be achieved. With this resolution, the QCM sensor can detect SoC changes in the battery of about 0.1% with a measurement interval of 2 s.

### 4.2. QCM Sensor in Lead-Acid Battery. “In-Situ” Resolution and SoC Measurements in Charge and Discharge Processes

**Figure 10.**
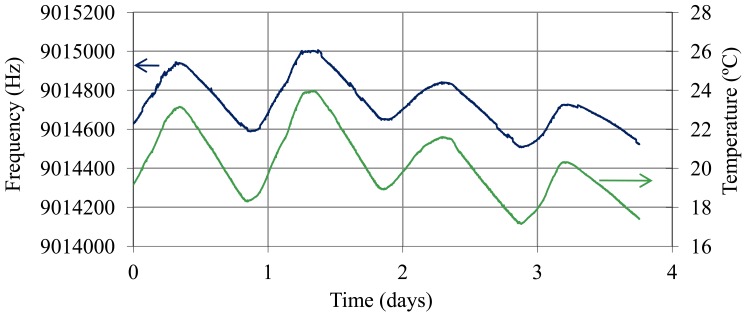
Oscillation frequency over four days with the resonator immersed in the battery cell of a fully charged battery (40% sulfuric acid) and temperature of the electrolyte.

**Figure 11.**
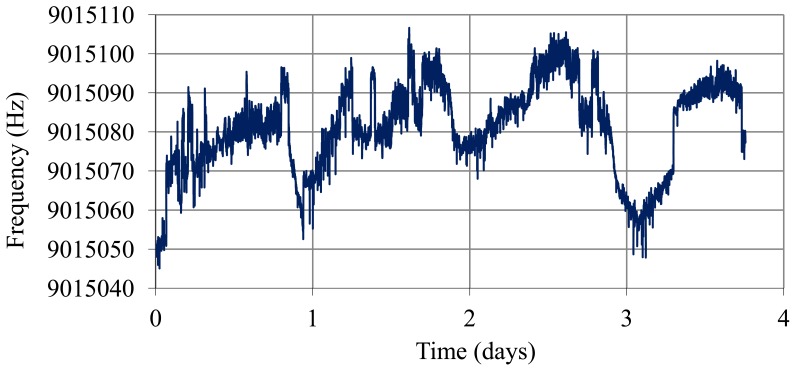

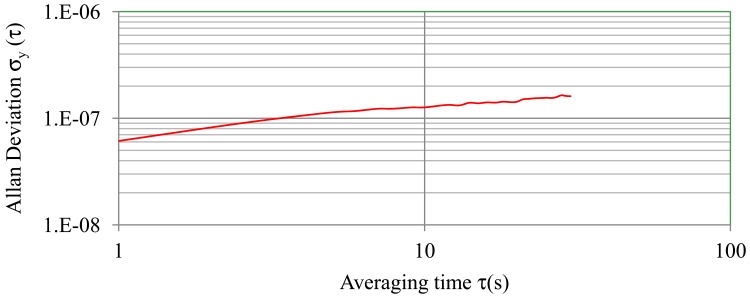
Oscillation frequency over four days after temperature compensation and Allan deviation.

Figure 10 shows the oscillation frequency of the sensor with the quartz resonator placed inside a fully charged battery (40% sulphuric acid) and the temperature of the electrolyte (battery at room temperature). Figure 11 shows the frequency values obtained after temperature compensation using Equations (14)–(17) and the Allan deviation for an averaging time from 1 to 30 s. The worst Allan value is 1.6 × 10^−7^ and therefore the resolution value is $8.5\times 10^{-4}\sqrt{\frac{g}{cm^{3}}}cp$. If we compare this “*in-situ*” noise values with values obtained in calibration with sulfuric solutions (Table 1), it can be concluded that frequency noise increases slightly when the resonator is placed inside the battery cell, perhaps due to temperature compensation. However, with this resolution changes can still be detected in the SoC of about 0.2% with a time interval of 2 s.

**Figure 12.**
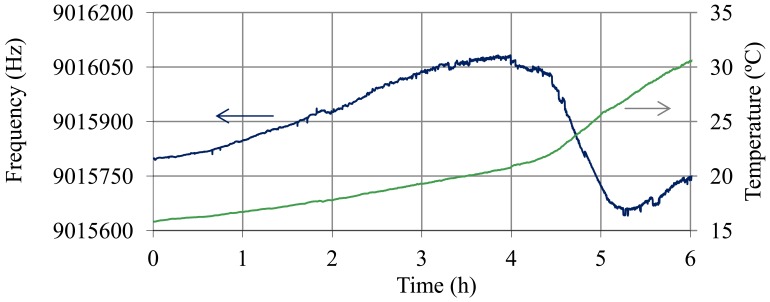
Oscillation frequency and temperature of the QCM sensor during a charge trial.

**Figure 13.**
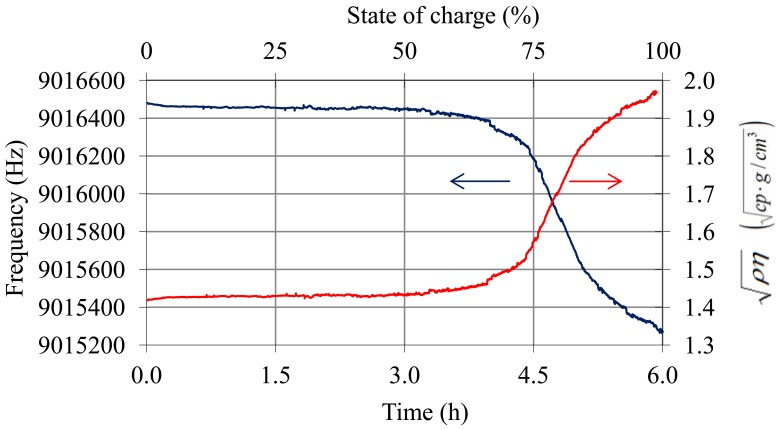
Frequency of the QCM sensor after temperature compensation using Equations (14)–(17) and variation of $\sqrt{\rho \eta }$ during the charge process.

**Figure 14.**
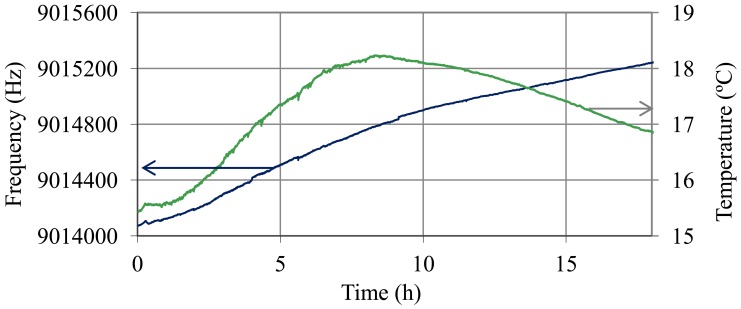
Oscillation frequency and temperature of the QCM sensor during the discharge trial.

**Figure 15.**
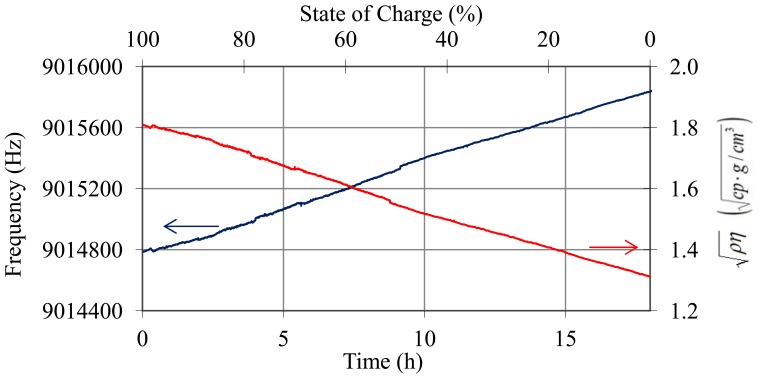
Frequency of the QCM sensor after temperature compensation using Equations (14)–(17) and variation of $\sqrt{\rho \eta }$ during the discharge process.

With respect to the tests in real-time charge and discharge processes, Figures 12–15 illustrate the results of the trials. Figure 12 shows the oscillation frequency and temperature of the battery during the charge trial. In Figure 13 the values of frequency after temperature compensation with Equations (14)–(17) are shown. After temperature compensation, the oscillation frequency depends only on the variation of viscosity-density product due to the shift of the SoC in accordance with Equation (1). The shift of $\sqrt{\rho \eta }$ measured by the QCM sensor is also shown in Figure 13. The sensor presents the expected tendency for the charge process in lead-acid batteries: in the top area of the battery, due to the electrolyte stratification, the density and viscosity do not increase their values significantly until the charge process is advanced and bubbling occurs [2,22].

During the discharge process there is no stratification and density-viscosity varies in a linear way with the state of charge. Figure 14 shows the oscillation frequency and temperature of the battery during the discharge trial. In Figure 15, the values of frequency after temperature compensation with Equations (14)–(17) and the resolved values for $\sqrt{\rho \eta }$ are illustrated.

An important aspect is the tolerance of the sensor to chemical attack from the sulfuric acid present in the battery electrolyte. The available literature on the chemical resistance of the materials which were to be in contact with the electrolyte was reviewed: quartz, gold (crystal's electrodes) and silicone (support's insulation coating). The quartz crystal holder is not in contact with the acid (it is coated with the silicone). It was discovered that these materials are immune to acid attack. However, there was uncertainty about the effect of acid on the chrome used as the adhesion layer for gold electrodes. To know this, the QCM resonator was immersed inside the battery cell for seven months. At the end of the trials, passive measurements of the quartz resonator equivalent circuit parameters were taken. Table 2 shows the measurements taken before beginning the trials and those made after the sensor had been inside the battery for seven months. In both cases, the measurements were taken with the crystal mounted in the home-made support. Based on these results, it is possible to consider that the sulphuric acid concentration present in the electrolyte is not aggressive for the sensor. Although R changes its value (8.88–11.05 ohms), this change is not significant and can be due to the fact that the cleaning conditions of quartz are not the same before and after the immersion of the sensor inside the battery. Before inserting the sensor into the battery, quartz has been thoroughly cleaned with acetone and distilled water. Then, the insulation silicone is deposited and when it is dry, electrical parameters of the sensor are measured. After seven months immersed inside the battery, the sensor is removed and carefully cleaned, but with only distilled water. Acetone is not used in order to avoid a chemical attack on the silicone, so it may be that the crystal has some impurity on the electrode when the electrical parameters of the sensor are measured again. There may also be a slightly aging of the sensor.

## 4. Conclusions

A high resolution QCM sensor for monitoring the state of charge in lead acid batteries was designed. Sensor calibration was done in sulphuric acid solutions with concentrations in the range of charge-discharge cycles. Frequency noise was determined, showing that there is a correlation between noise and $\sqrt{\rho \eta }$. Thermal testing of the quartz resonator in liquid media was conducted in order to develop a temperature compensated QCM sensor which avoids variations in the sensor response due to the usual electrolyte temperature variations that occur in battery charge and discharge processes. In this way, the relationship was obtained between the frequency shift and the variation of the square root of the density-viscosity product of the electrolyte due to temperature for a fully charged battery. Also, temperature compensation coefficients for the quartz immersed in the electrolyte of the fully charged battery were obtained. The thermal compensation of the two effects was employed successfully. Furthermore, the developed sensor was experimentally characterized in a battery and it was found to be capable of detecting changes in the sulphuric acid concentration of the electrolyte by means of changes in the square of the product density-viscosity with a resolution of $8.5\times 10^{-4}\sqrt{\frac{\text{g}}{{\text{cm}}^{3}}}\text{cp}$. Therefore, changes in the SoC of a battery of about 0.2% can be detected with a time interval of 2 s. Real-time tests were conducted during charge-discharge cycles of the battery to verify that the sensor responds appropriately. Also, the theoretical equation for the best QCM resolution for changes of the liquid density-viscosity product that can be achieved was obtained. This equation indicates that best resolution does not depend on the oscillator frequency. Hence, in order to improve the resolution, it is not necessary to increase frequency, but the noise present in the circuit must be reduced in order for real QCM sensors to approach the best resolution.

## Figures and Tables

**Table 1.** Density, viscosity, square root of the density-viscosity product, quality factor of the resonator charged with liquid, Allan deviation, detection limit, and resolution for each step of $\sqrt{\rho \eta }$.

***Step***	***ρ***	***η***	$\sqrt{\rho \eta }$	***Q****_exp_*	*σ_y_*(τ)	***Detection Limit***	***Resolution***
				
	$\left(\overset{g}{\;}/\underset{cm^{3}}{\;}\right)$	*(cp)*	$\left(\sqrt{\frac{g}{cm^{3}}cp}\right)$			*(Hz)*	$\left(\sqrt{\frac{g}{cm^{3}}cp}\right)$
1	1.096	1.331	1.208	1,795	1.51 × 10^−8^	0.1364	7.92 × 10^−5^
2	1.120	1.434	1.267	1,728	4.44 × 10^−8^	0.4000	2.32 × 10^−4^
3	1.143	1.544	1.328	1,659	3.61 × 10^−8^	0.3257	1.89 × 10^−4^
4	1.165	1.658	1.390	1,591	3.40 × 10^−8^	0.3067	1.78 × 10^−4^
5	1.186	1.777	1.452	1,526	3.08 × 10^−8^	0.2779	1.61 × 10^−4^
6	1.206	1.903	1.515	1,462	2.99 × 10^−8^	0.2692	1.56 × 10^−4^
7	1.223	2.024	1.574	1,408	3.67 × 10^−8^	0.3303	1.91 × 10^−4^
8	1.241	2.154	1.635	1,357	5.68 × 10^−8^	0.5117	2.97 × 10^−4^
9	1.258	2.286	1.696	1,313	6.49 × 10^−8^	0.5843	3.39 × 10^−4^
10	1.273	2.418	1.754	1,279	8.80 × 10^−8^	0.7923	4.60 × 10^−4^
11	1.288	2.554	1.814	1,253	5.61 × 10^−8^	0.5054	2.93 × 10^−4^
12	1.302	2.688	1.871	1,238	4.35 × 10^−8^	0.3920	2.27 × 10^−4^
13	1.316	2.824	1.928	1,233	4.16 × 10^−8^	0.3745	2.17 × 10^−4^

**Table 2.** Comparison between the EQC parameters of QCM sensor before beginning the trials in battery and after seven months immersed inside the battery's electrolyte. Measurements in air.

	**Before**	**After Seven Months**
*R (Ω)*	*8.88*	*11.05*
*L (mH)*	*6.44*	*6.40*
*C (fF)*	*48.37*	*48.68*
Cp (pF)	16.41	16.53
